# Spatial multi-scale relationships of ecosystem services: A case study using a geostatistical methodology

**DOI:** 10.1038/s41598-017-09863-1

**Published:** 2017-08-25

**Authors:** Yang Liu, Jun Bi, Jianshu Lv, Zongwei Ma, Ce Wang

**Affiliations:** 1 Business School, University of Jinan, Jinan, 250002 P. R. China; 2 State Key Laboratory of Pollution Control and Resources Reuse, School of the Environment, Nanjing University, Nanjing, 210023 P. R. China; 3 School of Geography and Environment, Shandong Normal University, Jinan, 250014 P. R. China

## Abstract

Adequately understanding the spatial multi-scale relationships of ecosystem services (ES) is an important step for environmental management decision-making. Here, we used spatially explicit methods to estimate five critical ES (nitrogen and phosphorous purifications, crop production, water supply and soil retention) related to non-point source (NPS) pollution in the Taihu Basin region of eastern China. Then a factorial kriging analysis and stepwise multiple regression were performed to identify the spatial multi-scale relationships of ES and their dominant factors at each scale. The spatial variations in ES were characterized at the 12 km and 83 km scales and the result indicated that the relationships of these services were scale dependent. It was inferred that at the 12 km scale, ES were controlled by anthropogenic activities and their relationships were dependent on socio-economic factors. At the 83 km scale, we suggested that ES were primarily dominated by the physical environment. Moreover, the policy implications of ES relationships and their dominant factors were discussed for the multi-level governance of NPS pollution. Overall, this study presents an optimized approach to identifying ES relationships at multiple spatial scales and illustrates how appropriate information can help guide water management.

## Introduction

Ecosystem services (ES) contribute to human wellbeing and have drawn considerable attention from governments and the public around the world^1, 2^. Numerous studies have been conducted to quantify, map, and value ES^3–5^. However, information on the relationships between ES remains limited, which presents challenges for integrating ES into actual management that considers the benefits for different stakeholder groups^6–8^. Previous studies have explored the ecological relationships among ES^9, 10^ as well as the congruence between ES demand and supply^11, 12^ at a given scale. Most results indicated that “trade-offs” and “synergetic” relationships occur among multiple ES or between different types of services, such as provisioning services, regulatory services, support services and cultural services^13, 14^. At the spatial and temporal scales and in terms of reversibility, ES relationships are complex^6, 15, 16^. Thus, further studies of ES relationships must be conducted at multiple scales.

Scale refers to physical dimensions in space and time, and is defined according to the extent and resolution^17, 18^. Ecological patterns and processes change at different scales, which is known as the scale dependence or scale effect^17, 19^. This feature is particularly apparent for spatial scales and represented by spatial heterogeneity as well as hierarchical variability. Ecosystems have different horizontal and hierarchical structures that determine ES spatial-scale features^20^ and further influence ES relationships^16, 21, 22^. Thus, identifying multi-scale spatial relationships among ES is important. Without an adequate understanding of these relationships, implementing management decisions may result in unexpected changes in the provisioning of ES and may threaten the stability and security of the ecosystems. However, to date, few ES studies have explicitly quantified ES spatial multi-scale relationships.

The scale dependence of ES relationships is not always apparent because of variations in methodological reliability as well as data availability and accuracy. Correlation coefficients, principle component analyses and overlap analyses have been commonly applied to examine ES relationships^12, 23–26^. Although these methods can analyse correlations in two or more ES, they cannot interpret the relationships at multiple spatial scales. Geostatistical methodologies have been widely used to model the spatial variability and correlations among heavy metals, nutrients, and physical-chemical properties in studies related to soil and the environment^27–29^. The factorial kriging analysis (FKA) method, which is a multivariate geostatistical approach, can examine the spatial relationships of given variables at multiple scales and divide the total variation into different spatial components that present separate relationships to the spatial scale^28^. The FKA method could lead to a better understanding of ES relationships at multiple spatial scales. To the best of our knowledge, classical geostatistical methods (e.g., ordinary kriging, indicator kriging, etc.) have rarely been used in the study of ES^30^, and FKA has never been applied to examine the spatial multi-scale relationships among ES. In this paper, we used FKA to quantify the spatial variability of ES and distinguish the static associations (positive or negative correlation) between ES at multiple scales.

Identifying the factors that influence ES is a key step in understanding the spatial scale dependence of ES relationships and essential to managing multiple ES to improve ecosystem functions^31, 32^. Nevertheless, the drivers of ES supply are complex, and the mechanisms underlying ES relationships are not straightforward^6, 12, 33^. Biophysical and socio-economic factors can be considered the main types of drivers that affect the spatial heterogeneity of ES^12^. These factors have scale-dependent impacts on ecological processes because of their different functional ranges^34, 35^ and drive changes in ecosystem functions, which result in variations in ES supply. Commonly, natural factors with wide spatial distributions may influence ecological processes over a large range, whereas human activities tend to operate at a smaller scale^27, 28^. Therefore, the relationships of ES are characterized by spatial scales and tend to vary from scale to scale.

Revealing the policy implications of spatial relationships among ES is essential for transferring ideas into public policy action and advancing sustainability goals^13, 16^. Recently, high-profile efforts have emphasized that ES relationships should be integrated into important social decisions^36^, including land use^25, 37–39^, ecosystem-based management^40, 41^, biodiversity conservation^32^, payment projects^42, 43^, etc. Nonetheless, limited information is available on quantifying ES relationships related to freshwater in a regional basin^13, 44^. In China, many watersheds suffer from critical issues, such as water quality degradation, water shortages, sediment erosion, and water use conflicts between upstream and downstream regions. Of all these issues, non-point source (NPS) pollution represents a primary problem. Thus, ES studies related to water pollution should be promoted for government policymaking^45^ (e.g., payment policy, spatially targeted management, etc.). Moreover, studies should be performed to identify sets of critical services, quantify services spatially and temporally, and understand their interactions and impact factors at different scales^6, 12, 16^.

Because of the demand for ES studies in regional watersheds, our study is designated to verify and interpret spatial multi-scale relationships of ES related to NPS pollution and identify the factors influencing regional water management. Thus, this study focuses on (1) quantifying five critical services (nitrogen and phosphorous purifications, crop production, water supply and soil retention) using spatially explicit methods; (2) using FKA to generate the spatial components for the ES relationships analyses at multiple scales; and (3) performing stepwise regression analyses to reveal the factors determining spatial multi-scale ES relationships. Moreover, we propose policy implications for managing NPS pollution from the perspective of ES multi-scale relationships. We selected the Jiangsu section of the Taihu Basin region as our study area because of its ecological and economic significance in China. We hope our study will provide information that can be used for freshwater management in the region and provide a basis for natural conservation area planning.

## Methods

### Study area and data materials

Taihu Lake is the third largest freshwater lake in China, and it is located in the highly developed and densely populated Yangtze River Delta of Eastern China^46, 47^(Figure S1). Taihu Basin covers 36900 km^2^. The Jiangsu section accounts for 53% of the basin and represents an independent water conservancy region divided by the Ministry of Water Resources of the P.R.C. We selected this region because it is a typical example of agricultural development that has occurred along with rapid urbanization in many parts of the world. The study area is densely populated (Figure S2) and is characterized by a diverse land use/land cover (Figure S3). NPS pollution and industrial pollution are the major environmental concerns in the region and have resulted in the serious degradation of freshwater ecosystems as well as frequent outbreaks of eutrophication since the 1990s^48^. Many measures have been implemented in China to improve water quality, such as major water-oriented special projects by the Chinese government. Currently, industrial pollution has been effectively controlled, although serious NPS pollution issues remain. Regional decision makers are attempting to balance agricultural development, urbanization and environmental protection. In this study, we analysed ES relationships and influencing factors to provide a reference for decision makers regarding NPS pollution. Data from 2010 were used because of their availability. Additional detail on the data sources is provided in the Supporting Information (SI text).

### Ecosystem services and factors quantification

We classified ES into direct services and indirect services based on the studies of Boyd *et al*.^49^, Johnston *et al*.^50^ and Nahlik *et al*.^51^. Excess nitrogen and phosphorus from NPS runoff has become the major threat to water quality in the study area. Therefore, nitrogen and phosphorus purifications were identified as direct services. Ecosystems provide water to generate hydrologic cycles that dissolve and separate out nutrients; therefore, the water supply could be selected as one indirect service. Soil retention was another indirect service because it can prevent soil erosion inputs to streams and maintain the soil’s capacity to filter pollutants. Crop production is an important provisioning service because of agricultural development in the Taihu Basin region. This service may aggravate NPS pollution through excess fertilization from farms; thus, we defined it as a negative indirect service.

A set of biophysical indicators were used as ES metrics as they can represent the ecosystem functions leading to human benefits and can be more easily understood by stakeholders and policymakers^13^ (Table 1). Nitrogen loading and phosphorus loading were inverse proxies of water purification services, which were different from three other indicators. We used biophysical models and empirical estimations to quantify and map the indicators of ES (SI text). Local monitoring, statistical data and similar researches were applied to evaluate the accuracy of the ES calculations. Details on the ES quantifications are specified in the SI text and Tables S1–S4.

**Table 1:** Ecosystem services and corresponding biophysical indicators.

Ecosystem Service	Biophysical indicator	Unit
Direct Service		
Water purification-Nitrogen loading (NL)	Annual nitrogen loading	kg/ha
Water purification-Phosphorus loading (PL)	Annual phosphorus loading	kg/ha
Indirect Service		
Water supply (WS)	Annual water yield	mm
Soil retention (SR)	Annual soil loss reduction	t/ha
Crop production (CP)	Annual crop yield	t/ha

Thirty-one environmental and socio-economic factors were selected for the dominant factor analysis after verifying the multicollinearity between factor variables using Spearman’s correlation^52^. We classified these factors into 10 types: climate, terrain, hydrology, soil, vegetation, accessibility, social development, residential condition, agricultural situation, and land use (see Table S5). The data at different resolutions were unified into a 30-m spatial resolution gird using ArcGIS 10.2 software (Esri, Inc.). Details of the factors quantifications are specified in the SI text.

### Spatial multi-scale analysis of ecosystem services

Because the 30-m spatial resolution raster complicated the following statistical analysis, we extracted the ES information from a subset of selected random points using ArcGIS 10.2 software. Different point numbers ranging from 1000 to 30000 in increments of 1000 were tested, and a value of 10000 sampling points was eventually selected by the cross-validation, which was the smallest value that resulted in a robust statistical analysis.

We used the FKA method to characterize the spatial variability and correlations of ES at multiple spatial scales. This method is used to fit a linear model of co-regionalization (LMC) and decompose total variation into multiple spatial components based on spatial structure ranges. The experimental variogram provides an empirical description of total spatial variability at different lag distances and is used to find key ranges of spatial structures. The variogram changes gradually with stepped increments in distance and appears to have an inflection point at one lag distance, which is the significant range of ES spatial variability. Once the range exceeds a certain distance, the variogram no longer changes, and this distance represents the maximum range of variability. Both ranges are key scales for dividing spatial components. Then, a structure correlation coefficient and principle component analysis (PCA) based on a co-regionalization matrix can be used to interpret the spatial interrelationships between variables at each scale^27^ and maps of spatial components can be generated via ordinary cokriging^53^. Detailed information on the FKA method is presented in the SI text. All steps of the geostatistical analysis were conducted using ISATIS software (Geovariances, Inc.).

In addition, we applied classical statistical methods, including Pearson’s correlations and PCA, to analyse the ES relationships without explicitly considering the different scales of spatial variability. These processes were performed using SPSS software (SPSS, Inc.).

### Identification of dominant factors

We used stepwise multiple regressions to identify the dominant factors that impact the spatial variation and ES relationships at each scale. The FKA estimates of the spatial components were considered dependent variables, and the influencing factors were considered independent variables. The R^2^ results from the regression models were used to explain the importance of the types of factors or individual factors. Stepwise multiple regressions were conducted in SPSS software (SPSS, Inc.).

## Results

### Spatial distribution of ecosystem services

**Figure 1:**
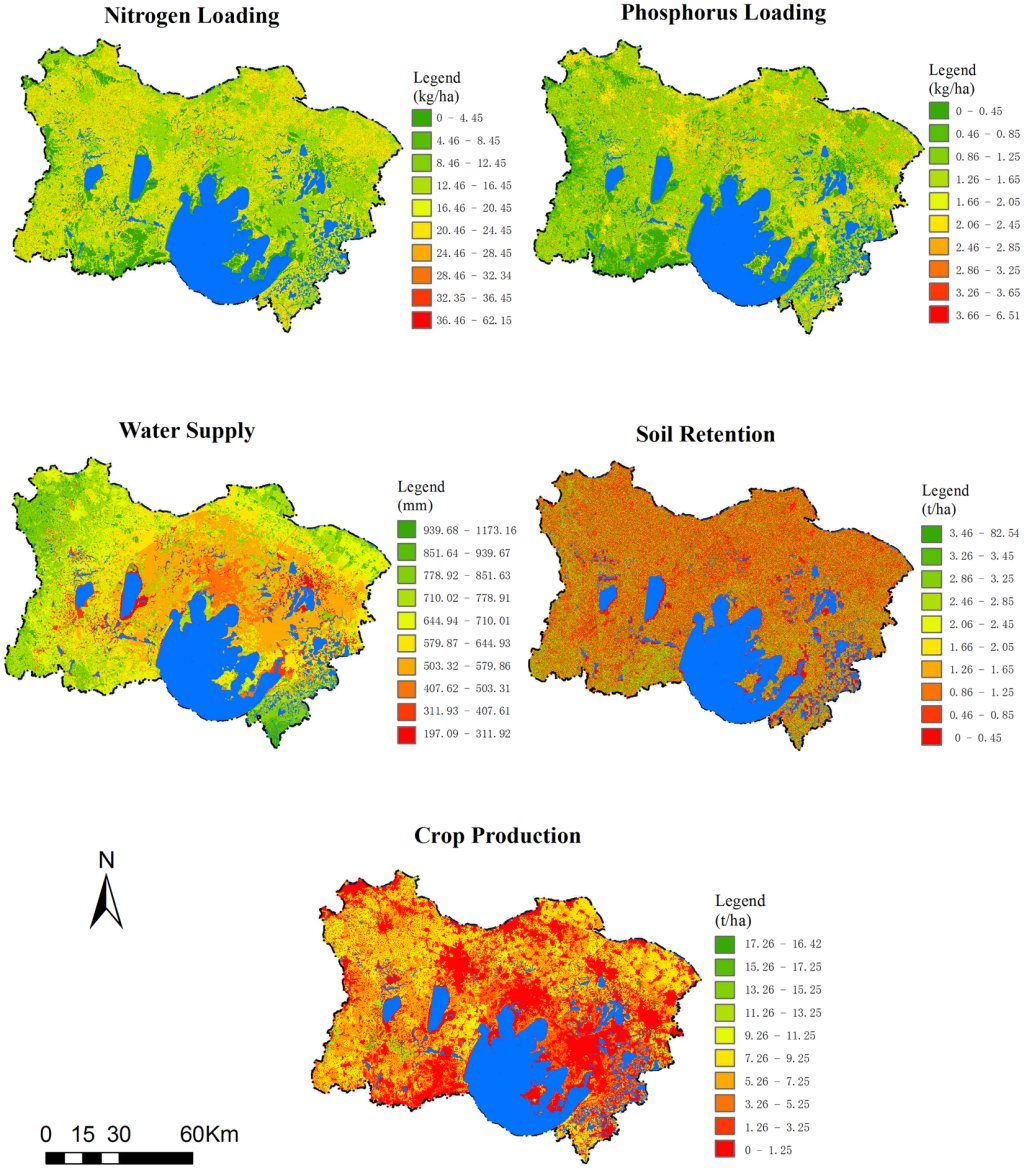
Spatial distribution of five ecosystem services in Taihu Basin, Jiangsu in 2010. Green indicates high value areas of ecosystem services, red and yellow indicate areas with low and moderate values, respectively, and blue represents water. Maps generated with ArcGIS 10.2.2 (http://www.esri.com/software/arcgis).

Empirical statistical and biophysical models were used to quantify five ES, and the spatial pattern of each service was the integrated result of the variables used to calculate the ES indicator. The resulting spatial distributions and statistical information for the five ES are shown in Fig. 1 and Table S6 respectively. The nitrogen loading (NL) value ranged from 0 to 62.15 kg/ha. The low value areas for the nitrogen purification service (high value areas of NL) were primarily distributed in the northern and western regions of the study area, whereas the high value areas (low value areas of NL) were located in the southern and eastern regions. The phosphorus loading (PL) value ranged from 0 to 6.51 kg/ha. The low value areas for phosphorus purification (high value areas of PL) were mainly located in the eastern and northern regions, whereas the areas with high value (low value areas of PL) were consistent with forest, wetland and ecological protection areas as determined through a spatial overlay analysis with land use/land cover maps (Figure S3). The water supply service (WS) value ranged from 197.09 to 1173.16 mm and was characterized by low values in the central region and high values in the surrounding areas. The soil retention service (SR) value ranged from 0 to 82.54 t/ha, with the low value areas showing a wide distribution and the high value areas showing a limited distribution, primarily in areas covered with forest and grass (Figure S3). The crop production (CP) value ranged from 0 to 16.42 t/ha. Spatial patterns of CP varied noticeably across the total area, with the high values in the west and north and low values in the non-agricultural land areas (Figure S3).

### Spatial variability of ecosystem services

The experimental variograms of ES variables (Figure S4) indicates that the inflection ranges are between 10 and 15 km, and that the variograms flatten out at ranges exceeding 80 km. Considering the optimum mean error (ME) and mean of squared standardized errors (MSSE), we uniformly divided the ES variations into 3 spatial components via LMC fitting. The theoretic variogram in equations (1) and (2) was expressed as the sum of 3 spatial components, which included a nugget effect, an exponential structure with a range of 12 km (short-range structure at the local scale), and a spherical structure with a range of 83 km (long-range structure at the regional scale).

1 $${\gamma }_{ij}(h)={b}_{ij}^{0}+{b}_{ij}^{1}(1-{e}^{-\frac{h}{12}})+{b}_{ij}^{2}[\frac{3}{2}(\frac{h}{83})-\frac{1}{2}{(\frac{h}{83})}^{3}]\,0\, < \,h\,\le \,83\,km$$

2 $${\gamma }_{ij}(h)={b}_{ij}^{0}+{b}_{ij}^{1}+{b}_{ij}^{2}\,h\,\ge \,83\,km$$

where ${b}_{ij}^{0}$ is the nugget effect, ${b}_{ij}^{1}$ is the sill of the short-range structure, and ${b}_{ij}^{2}$ is the sill of the long-range structure. The LMC parameters and variation percentages for each structure are presented in Table S7. The spatial ranges of 12 km and 83 km showed distinct changes in the variograms of the five ES variables.

The nugget effect tends to originate from errors due to data at different spatial resolutions and from sampling distance^27^. However, inherent variability and heterogeneity of the ES dominated the different nugget effects of five ES in this study. The nugget effect explaining the 89.46% of the total variance of SR was higher than those for other ES (Figure S4 and Table S7). It could be seen that the SR values are mostly low, as was the inherent variability and heterogeneity of SR (Fig. 1). The values of the experimental variograms did not increase significantly with the increasing lag distances, and all values of the experimental variograms were close to 1 (Figure S4), which result in the higher nugget effect than other ES. As our study aimed to reveal potential ES relationships at multiple spatial scales, thus the nugget effect was not considered in the subsequent analyses.

**Figure 2:**
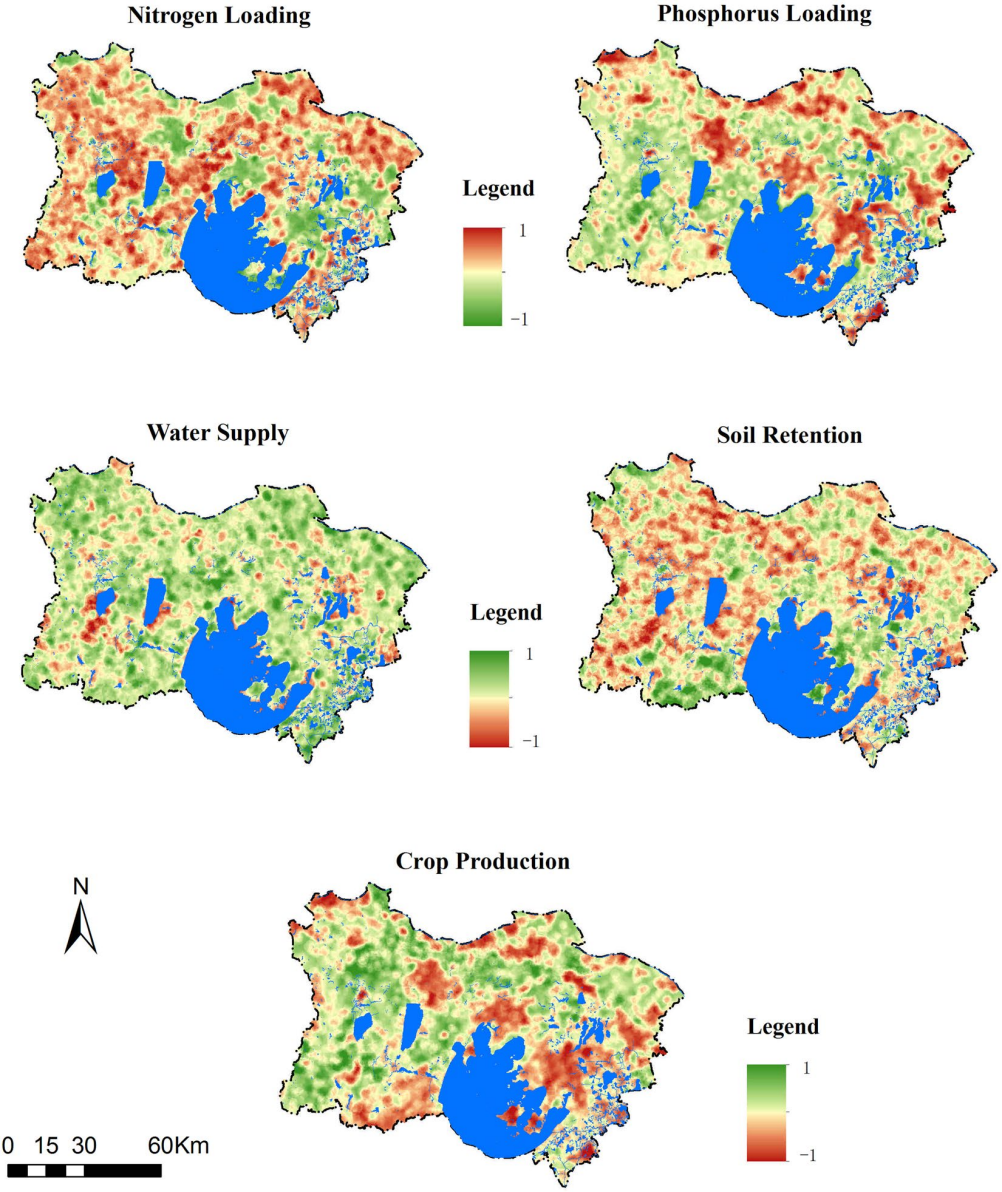
Spatial components of five ecosystem services at the local scale (12 km) in Taihu Basin, Jiangsu in 2010. 1 in the legend indicates high value areas of spatial component variability, −1 in the legend indicates areas with low value variability, and blue represents water. Maps were generated with ArcGIS 10.2.2 (http://www.esri.com/software/arcgis).

**Figure 3:**
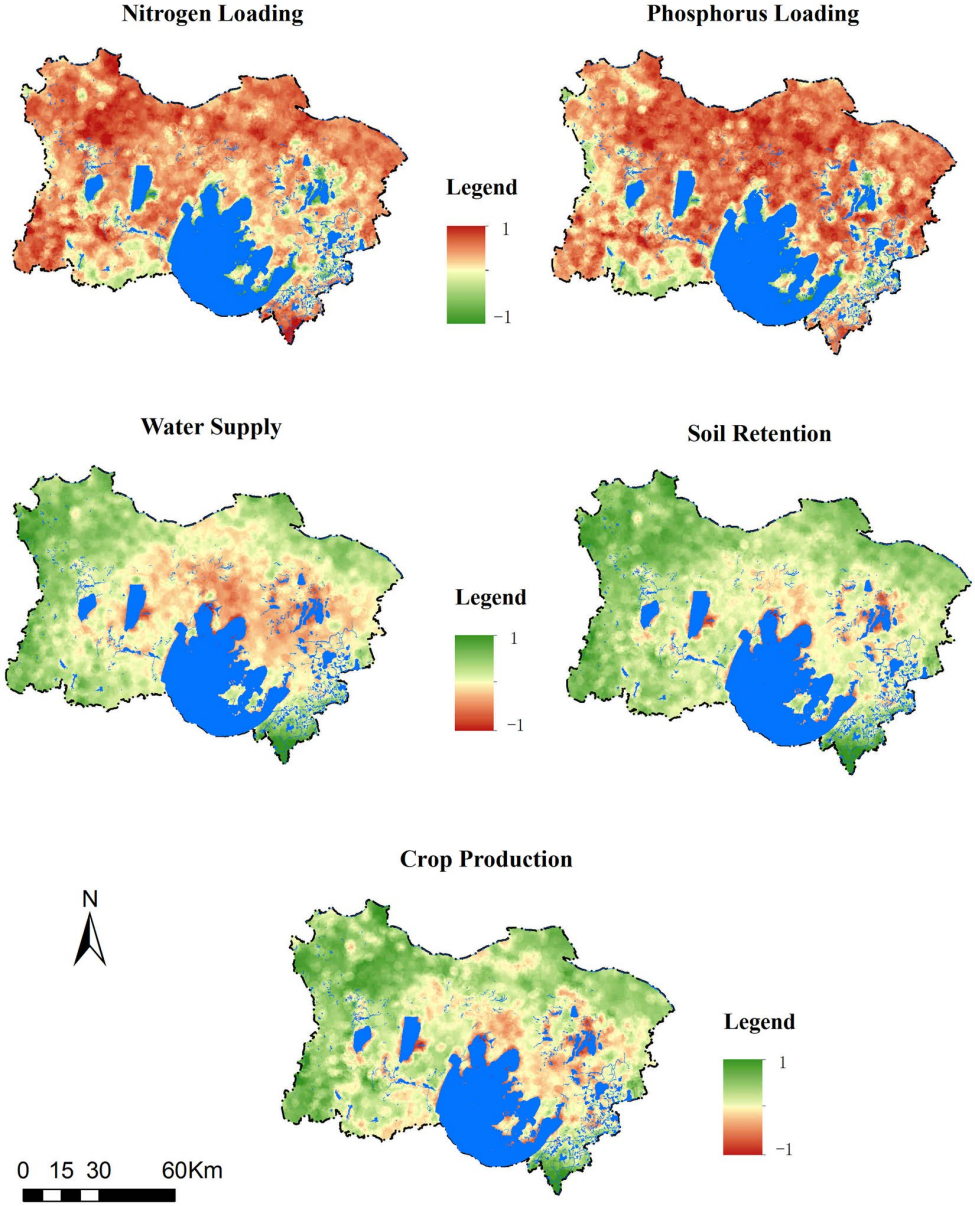
Spatial components of five ecosystem services at the regional scale (83 km) in Taihu Basin, Jiangsu in 2010. 1 in the legend indicates high value areas of spatial component variability, −1 in the legend indicates areas with low value variability, and blue represents water. Maps were generated with ArcGIS 10.2.2 (http://www.esri.com/software/arcgis).

The estimated spatial components of ES variations at the two scales were interpolated and mapped using ordinary cokriging with a 2D grid with 30-m mesh nodes^28^ (Figs 2, 3). The values of the spatial components ranged from −1.3 to 0.9. In order to show the spatial variability of ES explicitly, we standardized the original values of spatial components to a range between −1 and 1 at each scale. A value of -1 indicated the minimum value of ES components, and a value of 1 indicated the maximum value.

At the local scale, spatial variations in the ES components were relatively dispersed compared with ES variations in the regional scale. The patches with high values of NL, PL and CP were consistent with their spatial patterns (Figs 1, 2), whereas the high values of WS appeared to be random. At the regional scale, the spatial component variability of the ES presented a wide and continuous distribution. The NL and PL values exhibited similar spatial variations, and high values appeared to be more widely distributed than their corresponding spatial patterns (Figs 1, 3). The CP and SR values also presented similar variations and a small proportion of low values. The spatial component of the WS at this scale was consistent with its corresponding service pattern.

### Multi-scale relationships of ecosystem services

Pearson correlation coefficients were used to analyse the relationships of various ES (Table 2), with spatial variability indicated in Fig. 1. Structure correlation coefficients were calculated to analyse ES relationships at 12 km and 83 km scales (Table 2, Figs 2 and 3). Moderate correlations were observed between the NL and PL values (r = 0.526) by Pearson correlation analysis, and their correlations (r = 0.875) at the 83 km scale were stronger than those at the 12 km scale (r = 0.405). Pearson correlation coefficient of NL and CP was 0.491, and structure correlation coefficient was 0.702 and 0.786 at 12 km and 83 km scales respectively. PL had low correlation with CP at the 12 km scale, but a stronger relationship at the 83 km scale. WS had a high correlation with NL (r = 0.607) and PL (r = 0.735) at the 12 km scale, while the correlation weakened at the 83 km scale. Although generally weak correlations were observed between SR and other services at the 12 km scale, strong coefficients were observed at the 83 km scale. Overall, NL and PL showed positive correlations with other services, which revealed potential trade-offs between water purification services and other services. The structure coefficients mainly increased with increases in spatial scale, which suggests that correlations among ES were dependent on the spatial scale.

**Table 2:** Results of the Pearson and structure correlation coefficients among ecosystem services.

	Ecosystem service	NL	PL	CP	WS	SR
Pearson correlation coefficient	Nitrogen loading (NL)	1	**0.526**	**0.491**	0.595	**0.126**
	Phosphorus loading (PL)		1	0.058	0.510	0.007
	Crop production (CP)			1	0.509	0.171
	Water supply (WS)				1	0.145
	Soil retention (SR)					1
Structure correlation coefficient (12 km scale)	Nitrogen loading (NL)	1	**0.405**	**0.702**	**0.607**	**0.262**
	Phosphorus loading (PL)		1	−0.099	**0.735**	0.599
	Crop production (CP)			1	0.237	−0.091
	Water supply (WS)				1	0.566
	Soil retention (SR)					1
Structure correlation coefficient (83 km scale)	Nitrogen loading (NL)	1	**0.875**	**0.786**	**0.586**	**0.763**
	Phosphorus loading (PL)		1	0.679	**0.454**	0.652
	Crop production (CP)			1	0.707	0.840
	Water supply (WS)				1	0.772
	Soil retention (SR)					1

**Figure 4:**
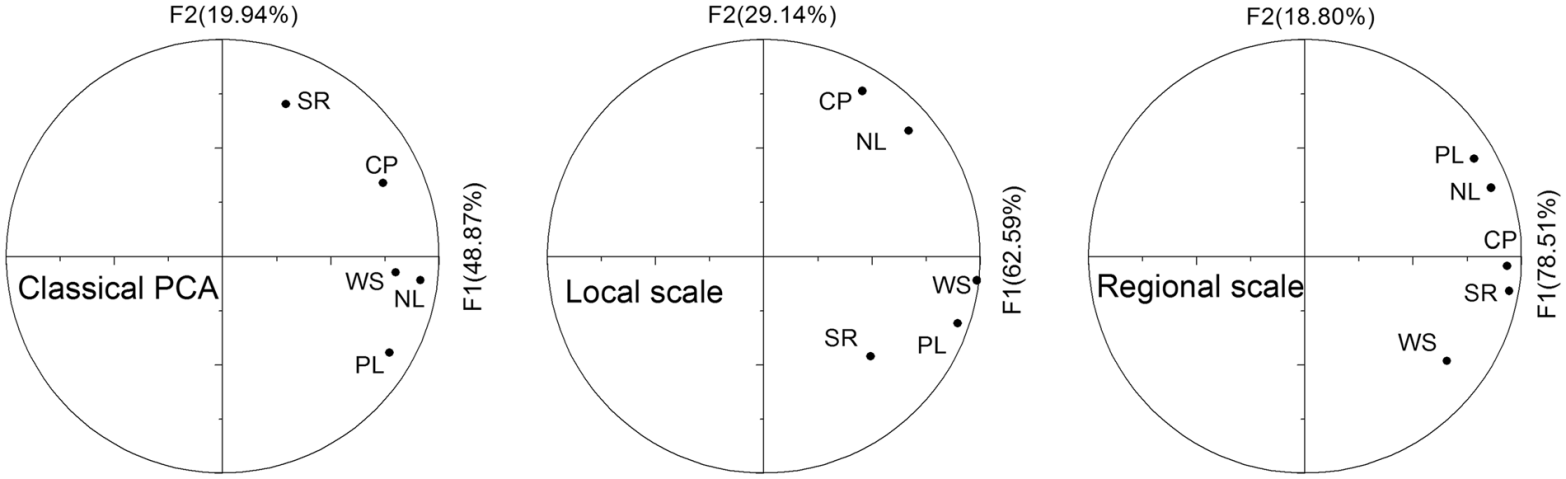
Correlation circles of principle component analysis for classical, local and regional scales.

The potential relationships among the five ES were further explored resorting to PCA at each scale (Table S8, Fig. 4). Classical PCA indicated that the first two components accounted for 68.81% of the total variance. The first component (F1) was positively related to the NL, PL, WS and CP values; and the second component (F2) was mainly explained by the SR values. At the local scale, 62.59% of the structural variance was explained by F1, which showed highly positive relationships between PL and WS. F2 accounted for 29.14% of the variance and showed highly positive relationships between CP and NL values, suggesting potential synergy between these variables. At the regional scale, F1 accounted for 78.51% of the structural variance and had strong and positive correlations with CP, SR, NL and PL, which indicated that synergies may occur among these variables. F2 accounted for 18.80% of the regional scale variation, had moderate correlation with WS. The PCA also confirmed the results of the structure correlations, indicating that the ES relationships were dependent on the spatial scale.

In the classical analysis approach, distinct variations occurring at different spatial scales were averaged, and the results contain inherent errors because of the nugget effect^27^. The FKA method can filter out the nugget effect to reveal potential ES relationships. The positive correlations between NL and CP were enhanced at the local and regional scales when the nugget effect was filtered out. The relationship between NL and SR was strong at the 83 km scale despite a lack of correlation by Pearson correlation analysis and at the 12 km scale. The spatial multi-scale relationships of ES in our study are similar to those of previous studies in other regions^13, 25, 30^, particularly the negative relationships between nutrient purification services and CP as well as SR at the regional scale. Overall the spatial multi-scale analysis of ES could better elucidate the underpinning spatial relationships of various ES by focusing on the structural components at different scales.

### Dominant factors analysis

The R^2^ results of the regression models showed the factors that influenced the spatial variations and relationships of ES at the two spatial scales (Table 3). The goodness of fit for the regression models was greater at the regional scale (66.8–74.2%) than the local scale (42.4–64.6%). Significant levels were observed for all regression models at p < 0.01. The influencing factors embodied in the regression models showed distinct differences with increases in spatial scale (Tables 3, S9 and S10), and ES grouped in the same principle component had similar regression models.

**Table 3:**
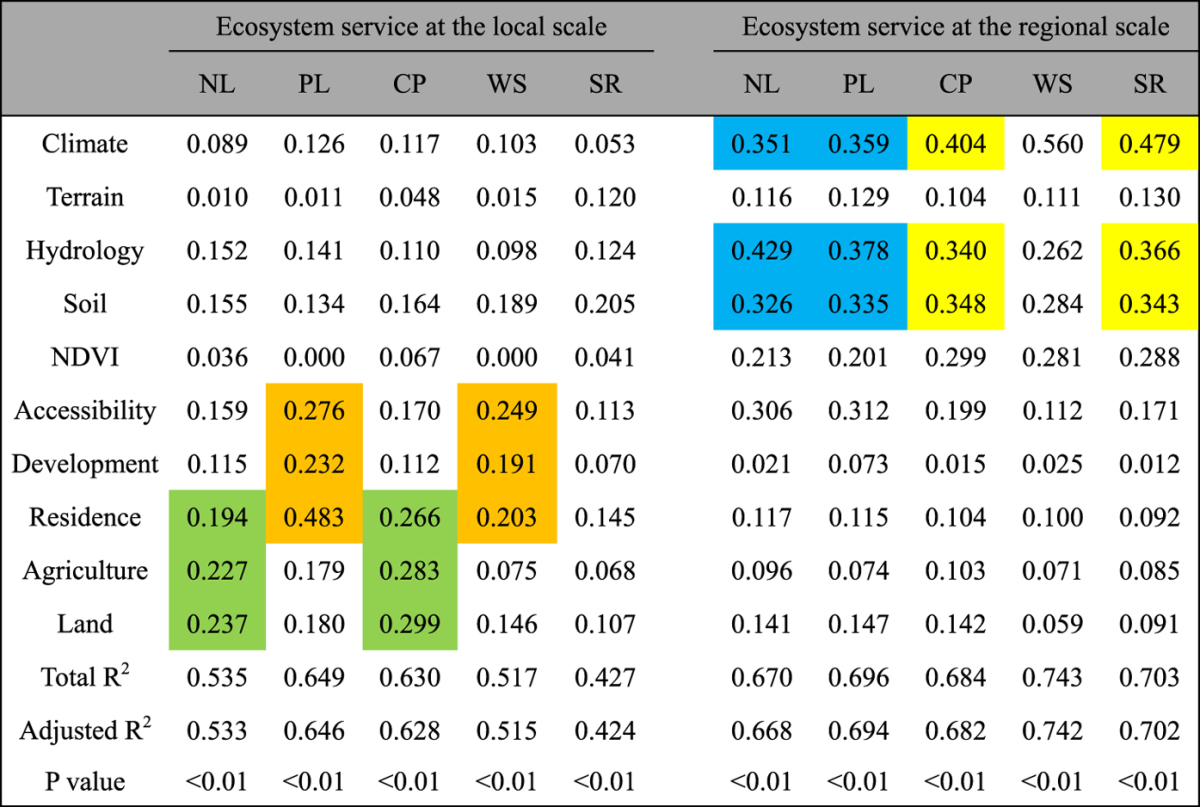
The R^2^ results of stepwise multiple regressions for ecosystem services and influencing factors at the local and regional scales. Ecosystem services with similar dominating factors are marked by the same colour.

At the local scale (12 km), socio-economic and accessibility factors played dominant roles in the spatial variations of ES and displayed generally higher R^2^ values than the other factors (Table 3). Spatial variations of NL and CP were primarily explained by factors of residence, agriculture and land use. For PL and WS, accessibility, development and residence factors mainly controlled the relationship. SR was primarily explained by soil, hydrology and residence factors, and these factors were different from the factors that explained the other ES; thus, SR was an isolated element in the PCA (Fig. 4). At the regional scale (83 km), physical and environmental factors (such as climate, hydrology and soil) could explain spatial variations of ES because their R^2^ values were distinctly greater than those of the other factors. For NL and PL, the dominant factors were hydrology, climate and soil, and other factors had similar contributions. Moreover, the spatial variations in CP and SR at the regional scale were primarily controlled by climate, hydrology and soil, but the influences of other factors were different from those that influenced nutrients loadings. For WS, the impact of climate was more notable than it was for the other ES; thus, WS was relatively independent in the PCA analysis at the regional scale (Fig. 4).

The factors that influence spatial variations in nutrient purification services must be identified to manage NPS pollution. At the local scale, NL was mainly attributed to the positive impacts of arable land, agricultural gross domestic product (GDP) and farmer income as well as the negative influence of water density and village density (Table S9). Intensive agricultural activities, particularly the excessive application of chemical fertilizers, have caused nitrogen enrichment and influenced ecosystem functions related to nitrogen purification. The excess nitrogen is transported by surface runoff, which results in the eutrophication of water bodies in the basin^46, 54, 55^. For PL, urban density, population and distance to water were the primary factors that had higher positive influence, whereas the distance to urban area and arable land had the most important negative effects. Previous studies also demonstrated that PL was closely correlated with urban land and non-agricultural industries^55–57^, and indeed the local patterns of PL (Fig. 2) are coincident with built-up areas (Figure S3). In the urban area, human activity is more prominent and leads to intensive land use and fragile vegetation cover, which reduce soil functions related to phosphorous purification^58^. In underdeveloped areas, garbage and excreta dumping and deficiencies in the collection and treatment facilities for sanitary sewage cause pollution as well as a high discharge of phosphorous into water, which influences the purification functions of the ecosystem.

At the regional scale (Table S10), water density was the most important negative factor for both NL and PL. Climate and soil factors also had important effects, and the distance to villages and urban areas had a negative impact on NL and PL respectively. Although the influence of terrain factors was enhanced at the regional scale, it played a limited role in the spatial variation of nutrients loadings.

## Discussion

### Scale dependence of ecosystem services relationships

Scale is a fundamental attribute that explains the patterns and processes of ES^31^, and both the perceived effectiveness of ES provision and delivery are influenced by scale^59^. In previous studies, spatial scales have commonly been treated as the spatial resolution of the available data as well as the spatial extent of the study region. Most studies have focused on modelling ES at a certain scale, including field, regional and national scales. Comparisons of ES and their relationships at different scales or across scales are rare. Norton *et al*.^60^ found that it was important to consider scale when developing ES indicators because scale influences the types of data and the process of gathering data. Similarly, a study by Anderson *et al*.^61^ indicated that the relationships between ES and biodiversity were sensitive to the data quality or the region size, finer resolutions and weaker correlations. Moreover, the relationships tended to shift systematically as the spatial extent of the analyses varied, which occasionally led to diametrically opposing conclusions. Emmett *et al*.^59^ analysed ES supply relationships at 1 × 1 km gridded and sub-catchment scales and found a similar result at both scales. Turner *et al*.^62^ analysed 11 ES in Denmark and found two scales (50 km and 150 km) of ES aggregation. Yang *et al*.^63^ used ES bundles to detect relationships of multiple ES at a city cluster scale, and the results indicated that fewer trade-offs and more synergies were observed among ES at a larger scale than a smaller scale.

Compared with previous studies, we used the FKA approach to illustrate the spatial-scale dependence of ES. This method is different from a grid-based approach in that sampling points are treated as spatially correlative in kriging algorithms and the correlations among points are reduced as the spatial distance increases. The variogram separated by a specific lag distance can be used to measure the spatial variability of any environmental variable. Increased attention should be paid to the distinct changes in variability of the lag distance of inflection in the variogram (Figure S4) because certain factors at this scale may fundamentally influence the spatial variability and relationships of ES. In this paper, we identified two key spatial scales: the local scale of 12 km and the regional scale of 83 km. The results indicated that the spatial variations and relationships of ES were dependent on the spatial scale. Weak correlations were observed between PL and CP without explicitly considering the different scales of spatial variability, whereas this correlation became slightly negative at the local scale (r = −0.099) and moderately positive at the regional scale (r = 0.679). A similar result was observed between CP and SR. The PCA performed at multiple scales also confirmed the importance of taking into account explicitly different spatial scales.

Different causes for the spatial-scale dependence of ES have been identified in previous studies. One explanation indicates that ecological processes operate across a range of scales and drive the diversity of ecosystem functions, thereby impacting the supply of ES at different scales^22^. Another explanation is based on the niche complementary effect in ecology^64^ in which a multi-species mixture can fully exploit a resource to achieve higher productivity compared with that of a monoculture. Similarly, the study units at the larger scale could carry more ES than those at the smaller scale^63^, thereby leading to scale effects in ES relationships. Moreover, unaccounted factors may influence ES relationships at local scale, whereas these factors can be overlooked when aggregating small units across areas at the regional scale^59^.

We used stepwise multiple regressions to analyse the manifold factors influencing spatial variability and relationships among ES variables at each scale. The results indicated that the ES controlled by similar factors tended to exhibit strong correlations at each scale. Socio-economic factors played a main role at the local scale, and interacted to impact the relationships of NL and CP as well as PL and WS. Intensive agricultural activities promote CP and increase NL through the application of excessive nitrogen fertilizer. Local industry production and domestic sewage pollution facilitate phosphorus discharge, influence land cover and surface runoff, and change the water supply in the ecosystem. At the regional scale, physical environmental factors (such as climate, hydrology and soil) controlled ES relationships and caused them to display positive correlations. The geographical signature determines the background conditions and influences the efficiency of human activities^35^. Local socio-economic factors can be overlooked at a larger scale, thereby reducing the spatial heterogeneity of ES. Drivers of environmental change exhibit scale sensitivity^35^, and although anthropogenic drivers can operate at multiple administrative scales, the impact of human activity tends to attenuate across scales^65^. On the whole, physical environments generally determine the fundamental characteristics of ES supply and control their general spatial distributions, whereas socio-economic factors influence local scale ecological processes^24^ and result in higher spatial heterogeneity of ES.

### Policy implications for controlling non-point source pollution

Understanding the effects of different spatial scales is necessary for measuring and managing ES. The diversity of findings suggests that additional attention should be focused on regulatory services at regional scales and provisioning services at local scales^31, 66^. Research shows that most ES should be managed mainly at the individual patch scale of land use, whereas ES closely related to human uses can be managed at the municipal scale^32^. Our work revealed that scale dependence is associated with ES variability and relationships, and the policy implications of our results are described as follows. First, the importance of spatial scales must be recognized to synergistically manage multiple ES. In our study, diverse spatial correlations were observed among the ES within a range of 12 km and additional synergy was observed beyond 83 km. Scale plays an important role in identifying ES relationships; therefore, decision makers should identify the most relevant scale for collecting data and evaluating ES to implement synergetic policies.

Second, multi-level governance should be implemented because of differentiated ES relationships and dominant factors at multiple scales. Generally, scattered variations at the local scale provide information for decision making performed at lower-level administrative regions, whereas continuous variations at the regional scale play a more important role for higher-level administrative regions. The administrative divisions in China are organized into five practical levels: provincial, prefectural, county, township, and village levels (from high to low). The spatial scales derived from the FKA are the same as the equivalent diameters of circular regions and can be linked to administrative scales by comparing the areas of spatial ranges and administrative regions. In the present study, the spatial scale of 12 km most closely aligns with the township level, whereas the 83 km scale could match the prefectural level in the study region. Related policies should be addressed at the township and prefectural levels. Stakeholders at different levels often evaluate ES distinctively, and they cut across a range of institutional scales^21^. Our results can facilitate public perception at both scales, promote targeted public actions for the local or regional contexts, coordinate multiple ES, and optimize comprehensive benefits for the entire region.

Furthermore, policymakers should implement regionalized management that integrates ecological and administrative scales. In our study, the ranges generated by the variogram fit represent the ecological scales of the ES relationships. However, the ecological scales of environmental problems rarely coincide with the governmental scales of decision making and management^21, 34, 67^. Thus, ES management should consider the differences between the ecological scales of ES relationships and the governmental scales of decision making^34^. Spatial-scale analyses based on the FKA approach could diminish this gap by comparing the spatial range with the administrative region. Additionally, updating partitions to fit ES management can reduce the inconsistencies between administrative divisions and ecological scales.

Specifically, the results provide information for controlling NPS pollution at the given spatial scales. At the local scale, human activities, which represent the dominant factors controlling ES variations and relationships, could be regulated to improve ecosystem functions and reduce NPS pollution. For nitrogen related services, reductions in arable land and agricultural activities (based on influencing factors identification) are effective methods of regulating nitrogen pollution. However, according to the remarkably positive relationship between NL and CP, reductions in arable land and agricultural activities will reduce CP and threaten important services for regional food security. Therefore, policies for nitrogen control should consider the full spectrum of factors related to water quality protection and agricultural development. Although several management policies have been conducted in pilot regions of the study area, including organic farming, ecological compensation, and soil testing for formulated fertilization, their effects have been limited^68, 69^. We suggest that these policies might be optimized by considering ES relationships and effective spatial scales. For phosphorous service, PL was closely related to urban density and distance to urban areas, thereby demonstrating that urbanization may influence ecosystem capacity and cause increases in NPS pollution. Therefore, urbanization should be more rationally planned, and it is particularly important that urban infrastructures and landscapes should be strengthened in urban planning. Overall, the relationships and dominant factors of nutrients loadings at the 12 km scale suggest that simultaneously optimizing nitrogen and phosphorous services is impractical and indicate that policymakers should implement differentiating measures to control nutrients loadings at the town level. At the regional scale, the variations and relationships of NL and PL were dominated by physical environmental parameters (such as climate, hydrology and soil), and climate change and subsequent hydrological processes could alter the inherent ES supply and relationships. Under the climate change conditions, adaptive strategies^70^ for managing multiple ES should be concentrated at the prefectural level. Furthermore, distance to village and urban areas negatively influenced the NL and PL values, respectively. Therefore, these services could be improved by reducing the residential density, and land development planning should be strengthened to maintain ecological security patterns at the prefectural level.

### Advantages and limitations

Multi-scale spatial analyses represent a challenge for ES studies^71^. Because of the complex mechanisms underlying ES scale dependency, a classical approach cannot interpret the significant potential relationships among ES. The combination of a FKA and regression analysis provides a powerful approach to quantifying and explaining the multi-scale relationships of ES and has been applied in the case study of the Taihu Basin region. The FKA method could effectively decompose the total variation of ES into multi-scale spatial components and quantify ES relationships at each given scale. The regression model provides a good reference for quantitatively identifying dominant factors and provides important insights on the mechanisms influencing ES relationships. Our results indicated that ES relationships present a scale-dependent feature and anthropogenic activities influenced differences in ES relationships at the 12 km scale while physical environments (such as climate hydrology and soil) had a dominant effect on ES relationships at the 83 km scale.

The sensitivity of ES relationships depends on the accuracy of ES quantifications and indicators selection. Inconsistencies in the resolution of spatial data from multiple sources along with the uncertainty in the parameters used to calculate each indicator represent the primary uncertainties and challenges for studies quantifying ES and determining their relationships. Data at a coarse resolution may dominate data at a fine resolution during the calculation process, which would decrease the accuracy of the final ES results^22^. The FKA method can filter out the nugget effect and identify useful factors at multiple spatial scales; therefore, this method can manage inconsistencies among data at multiple resolutions. However, such data inconsistencies tend to result in a high percentage of nugget effects in LMCs (Table S7). Therefore, optimized methods should be proposed to resolve issues related to data inconsistencies and promote the effective percentage of total data.

Although spatially explicit methods were used to model the locations and spatial variations of ES supply, the spatial patterns of synergies and trade-offs among ES (such as “hotspot areas” of ES relationships^13^) were not included in our study. Moreover, the relationships among services may vary with changes in the temporal extent. Therefore, quantifying and mapping the spatial congruence of ES and modelling the dynamic trends in ES relationships will be considered in our future work. Although spatial variations in ES may be caused by similar factors, these ES are not necessarily driven by the same processes; therefore, additional biophysical and socio-economic drivers must be explored to clarify ES relationships.

## Electronic supplementary material

Supporting Information
