# Supplementary material for: Spatial multi-scale relationships of ecosystem services: A case study using a geostatistical methodology
Source: Sci Rep. 2017 Aug 25;7:9486. doi: 10.1038/s41598-017-09863-1 (PMC5573410; doi:10.1038/s41598-017-09863-1)
Supplement: Supplementary file 1 — Supporting Information [file 41598_2017_9863_MOESM1_ESM.pdf]

Spatial multi-scale relationships of ecosystem services: A case study using a geostatistical methodology

Yang Liu[1,2], Jun Bi[2]*, Jianshu Lv[3], Zongwei Ma[2], Ce Wang[2]

1. Business School, University of Jinan, Jinan, 250002, P. R. China.

2. State Key Laboratory of Pollution Control and Resources Reuse, School of the Environment, Nanjing University, Nanjing, 210023, P. R. China.

3. School of Geography and Environment, Shandong Normal University, Jinan, 250014, P. R. China.

* Corresponding author

E-mail: jbi@nju.edu.cn

Tel. /Fax: +86 2589681605

Supporting Information

Characteristics of the study area

The Taihu Basin includes the southern region of Jiangsu Province, northern parts of Zhejiang Province, most of Shanghai Municipality and a small section of Anhui Province (Figure S1). The basin is characterized by a warm moist monsoon climate. The annual rainfall and annual average temperature is approximately 1,180 mm and 17 ℃, respectively[1]. The study area in Taihu Basin is one of the most economically developed areas, with GDP accounted for 5.2% of China's GDP in 2010[2]. The study area is one of the most densely populated region, with 22,430 thousand inhabitants in 2010[2], and concentrated in cities and towns (Figure S2). The cultivated land, covering 45.97% of study area, is mainly located in the west and northeast area. The south mountain area is mostly covered by forest and grassland, accounting for about 5.55% and 0.13% of the study area, respectively (Figure S3).

Data acquisition

Climate data were derived from the China Meteorological Data Sharing Service System (http://cdc.nmic.cn/). Land use and road data were obtained from the National Earth System Science Data Sharing Infrastructure (http://www.geodata.cn). Digital elevation model data (DEM) and remote sensing (RS) image data with a resolution of 30 m × 30 m were downloaded from the International Scientific Data Service Platform (http://datamirror.csdb.cn/) and Geospatial Data Cloud Platform (http://www.gscloud.cn), respectively. Soil data with a resolution of 100 m × 100 m were provided by Nanjing Agricultural University and the Institute of Soil Science, Chinese Academy of Sciences[3]. Population density and GDP were provided by the Institute of Geographic Science and Natural Resources Research, Chinese Academy of Sciences. Hydrologic, water quality, agricultural and other statistical data were acquired from the local water conservancy, environmental protection and statistical departments of each county. Biophysical parameters were obtained from regional government documents and related publications, and the details are specified as follows.

Ecosystem services mapping

Water purification services

Eutrophication is one of the most serious problems in the study area[4]. Excess nutrients input, primarily nitrogen and phosphorus from non-point source runoff, has become a major threat to water quality. Ecosystems can purify water by removing nutrient pollutants from runoff via vegetation and soil, thus illustrating the terrestrial contributions to water quality. Annual nitrogen loading (NL) and phosphorus loading (PL) values are

used as proxies for water quality services, and lower loading values correspond to higher water quality services. We used the Nutrient Purification Model of Integrated Valuation of Ecosystem Services and Trade-offs (InVEST) to quantify the NL and PL[5, 6], and the major data inputs and parameters are listed in Table S1. The model can be expressed as equation (1).

$$Exp_x = ALV_x \prod_{y=X+1}^{X} (1 - E_y) \quad (1)$$

where Exp_x represents the nutrient exported from the upper grid to downstream water (kg/ha); E_y represents the filtration efficiency downstream at pixel *y* (%); *X* represents the nutrient transport route, which is a dimensionless parameter; and $\mathrm{ALV_x}$ represents the nutrient loading at pixel *x* (kg/ha), which can be calculated by equation (2).

$$ALV_x = HSS_x \cdot pol_x \quad (2)$$

where pol_x represents the nutrient export coefficient at pixel *x* and HSS_x represents the sensitivity value of hydrology, which is calculated by equation (3). In this equation, λ_x represents the runoff coefficient at pixel *x* and λ_w represents the average runoff coefficient of the entire region. The parameters for the nutrient export coefficient, filtration efficiency and flow accumulation threshold were derived from published field studies and local estimates[4, 7, 8].

$$HSS_x = \frac{\lambda_x}{\lambda_w} \quad (3)$$

More details can be found in the user's guide for the InVEST model[9]. For the assessment, the nutrient loadings were recalculated using the BASINS model for the Meilin and Lihe sub-watersheds. We also compared our results with several similar studies in the region[10-13].

Water supply service

Water supply service (WS) is the surficial water yield generated by the hydrologic cycle, and it provides water for human consumption or hydropower production. The WS is influenced by meteorological factors as well as soil properties and land cover. Considering the combined impact of multiple factors, we used the Water Yield Model of InVEST to quantify the annual WS[14], and the major data inputs and parameters are listed in Table S2. The equation for WS at each pixel *x* of landscape is expressed as follows in equation (4).

$$WS(x) = \left(1 - \frac{AET_x}{Pre_x}\right) \cdot Pre_x \quad (4)$$

where Pre_x represents the annual precipitation on pixel *x* (mm); AET_x represents the annual actual evapotranspiration (mm); and $\frac{AET_x}{Pre_x}$ is expressed based on the Budyko curve[15, 16] as shown in equation (5).

$$\frac{AET_x}{Pre_x} = \frac{(1+\omega_x+R_x)}{(1+\omega_x+{}^{1}/_{R_x})} \quad (5)$$

where ω_x represents a non-physical parameter and is calculated by equation (6), and R_x represents the aridity index for pixel *x* and is calculated by equation (7).

$$\omega_x = Z \times \left(\frac{AWC_x}{Pre_x}\right) \qquad (6)$$

$$R_x = \frac{k_j \times ET_0}{Pre_x} \qquad (7)$$

In equation (6), Z is an empirical constant that captures the precipitation pattern and hydrogeological characteristics, AWC_x is the volumetric plant-available water content (mm) estimated by the minimum of the root-restricting layer depth (*Rest.layer.depth*), vegetation rooting depth (*Root.depth*) and plant-available water capacity (*PAWC*). *Rest.layer.depth* (mm) and *PAWC* (mm) were provided by Nanjing Agricultural University and the Institute of Soil Science, Chinese Academy of Sciences[3]; the *Root.depth* parameter (mm) for each land type was assigned a value according to the guide book of InVEST and the research results of Canadell *et al.*[17], Huang *et al.*[18], Liu *et al.*[19], and Lv *et al.*[20]. In equation (7), k_j is the evapotranspiration coefficient (%) of each land use/land cover based on the guide book of InVEST as well as the research results of Gao *et al.*[21], Ji *et al.*[22], and Lv *et al.*[23], and ET_0 (mm) was quantified by the Modified-Hargreaves Method.

The performance of the Water Yield Model was tested in the Meilin and Lihe sub-watersheds, which have dense and consistent hydrological monitoring data from the last decade. The water yield results largely depend on the accuracy of the input data[24], and a sensitivity analysis showed that the annual precipitation is the primary sensitivity factor in the study area. Therefore, we used different interpolation methods to model the spatial distribution of precipitation, including an inverse distance weight method (IDW), spline function method (Spline), kriging interpolation and Thiessen polygon methods. By comparing model data with observation data in two sub-watersheds which have intensive monitoring stations, we selected kriging interpolation to produce a 30-m pixel raster for spatial predictions of precipitation. Our estimate was reasonably consistent with the averaged water yield of the sub-watersheds due to the Nash–Sutcliffe model efficiency coefficients ranged from 0.52 to 0.85.

Soil retention service

Ecosystems play an important role in preventing soil erosion inputs to streams and are associated with the soil's capacity to filter pollutants, which can regulate water quality. We quantified the annual potential reduction of soil loss as the indicator for soil retention service (SR) by referring to the Revised Universal Soil Loss Equation (RUSLE)[25, 26], and the major data inputs and parameters are listed in Table S3. However, RUSLE was developed to model soil erosion in the Midwestern U.S. and may generate deviations from the actual conditions

of the Taihu Basin region. Therefore, we adjusted the model using parameters based on experimental data from the sub-watersheds. The equation for the SR in this study can be expressed as equation (8).

$$SR = R \cdot K \cdot LS \cdot (1 - C \cdot P \cdot A) \qquad (8)$$

This model indicates that current land uses that implement vegetation cover and protection measures will generate reductions in soil loss compared with land uses that lead to bare soil. R is the rainfall erosivity index (MJ·mm·(ha·h·a)$^{-1}$), which was calculated by the empirical formula of Wischmeier *et al.*[27]; K is the soil erodibility (t·ha·h·(ha·MJ·mm)$^{-1}$), which was calculated by the EPIC (Erosion Productivity Impact Calculator) equation of Williams *et al.*[28]; LS is the slope length and steepness index, which is a dimensionless parameter quantified by the method of Liu *et al.*[29]; C is the cover-management factor, which is a dimensionless parameter with a range from 0 to 1, and it was quantified by the method of Lv *et al.*[30]; P is the support practice factor, and it ranges from 0 to 1 and was determined based on the study of Zhang *et al.*[31]; A is the adjustment factor for modifying the impact of uncertainty, and it was set as 1.3 in our study area.

Accuracy was assessed by comparing our estimates with field measurements of soil erosion in two sub-watersheds (Meilin and Lihe) as well as with the results of previous similar studies[32, 33] in the region. Overall, our estimates are consistent with the findings of previous studies in terms of the total value and spatial distribution.

Crop production service

The annual crop yield per unit area was used as an indicator for the crop production service (CP). The crop types in the study area include rice, wheat, corn, soybean, sorghum, millet, and potato, and they grow in different seasons. We converted the production of different crop types into the standard yield and estimated the total yield in a year using a climatic potential productivity model and cultivated land quality data of the study area, and the major data inputs and parameters are listed in Table S4. The cultivated land quality data can capture the integrated effect of local weather conditions, soil fertility, and other environmental factors. The equation for CP is expressed as equation (9):

$$CP = P_v \cdot g(x) \qquad (9)$$

where P_v represents the climatic potential productivity (kg(ha·a)$^{-1}$) which was calculated by the Thornthwaite Memoriai Model[34], and *g(x)* represents the adjustment value at each pixel *x*, which is a dimensionless parameter, which was obtained from cultivated land quality data. According to relevant regulations[35, 36] and expert knowledge, cultivated land quality data were divided into 22 levels and assigned different potential yield coefficients. After the conversion to a 30-m resolution, land use maps and potential yield

data were overlaid to estimate the crop yield in each pixel.

We assessed the accuracy of the estimated results at an aggregated level because actual data could be acquired for each administrative unit[6]. Compared with the actual yield for 2010 at the town level, our estimate showed good performance because the deviation was within 10.7% for the region. Furthermore, town-based actual data and an area-weighted method based on arable area were used to adjust the regional data if errors were greater than 5%.

Quantifying influencing factors

Physical environmental factors

Six groups of physical environmental factors were quantified (Table S5): (i) climate data (percentage of actual and potential sunshine duration, annual average relative humidity and annual average temperature) were obtained via an ordinary kriging interpolation of point data from 127 meteorological stations into 30-m resolution grids[37], besides data from 162 meteorological stations used for modelling precipitation; (ii) terrain data (altitude and slope) were measured based on a DEM with the 3D analysis module in ArcGIS (Esri, Inc.); (iii) water density as an important hydrological indicator was quantified through the water area in each 1-km grid; (iv) soil parameters (total nitrogen, total phosphorous, total potassium, organic matter, bulk density, and percentages of sand, silt and clay particles) were extracted from the soil attribute database and then transformed into raster data; (v) NDVI (normalized difference vegetation index) data were calculated from TM images using the ENVI platform; and (vi) accessibility factors (distance to water, cities and villages) were quantified by Euclidean distance in ArcGIS (Esri, In annual c.) based on raster data of construction land and water body.

Socio-economic factors

Economic, population, agricultural and residential conditions were included as socio-economic factors: (i) development factors (population density and GDP per area) were adjusted using the Areal Weighting Interpolation in Grid Cells method based on original raster data and current town data from the study data[38]; (ii) residential condition factors (density of roads, cities and villages) were acquired from the area in each 1-km grid of corresponding construction land; (iii) agricultural status data were available at the town level, of which agricultural population and GDP were standardized by the area of each town, the total power of agricultural machinery was standardized by the cultivated area of each town (in hectares), and the data on farmer's annual average income were derived from every town and set as homogenous at the town level; and (iv) Land use shares (arable, forest, grass and wetland) were expressed by area ratios for every square kilometre.

Factorial kriging analysis

Factorial kriging analysis (FKA), a multivariate geostatistical method, can model the multi-scale spatial variability of multiple variables. The variogram is broken down into several structures in FKA, and correlations between variables at each structure are described by a co-regionalization matrix. Overall, FKA primarily involves fitting a linear model of co-regionalization (LMC), identifying multi-scale spatial relationships between variables, and mapping the spatial components of variables at each given scale[39, 40]. To eliminate the presence of outliers, we used Gaussian anamorphosis to standardize the original variables into a Gaussian-shaped variable, and the FKA was conducted on the Gaussian-shaped variables[40].

First, the LMC is used to fit the m(m+1)/2 direct and cross-variograms of the m variables and can be written as the matrix term in equation (10), which means that the variogram $\Gamma(\mathrm{h})$ is decomposed into several spatial structures labelled by u:

$$\Gamma(h) = \left[\gamma_{ij}(h)\right] = \sum_{u=1}^{N_s} B^u g^u(h) \qquad (10)$$

where $\Gamma(h)$ is the variogram matrix of order m×m, *h* is the lag distance; $\gamma_{\mathrm{ij}}(\mathrm{h})$ is the cross-variograms between two variables *i* and *j*, which is expressed as equation (11); N_s is the number of the structure; B^u is the co-regionalization matrix, which describes the relationships between m variables at the given spatial scale *u*; and $g^u(h)$ is the basic variogram that represents variogram components for different spatial structures.

$$\gamma_{ij}(h) = \frac{1}{2N(h)} \sum_{\alpha=1}^{N(h)} [z_i(x_\alpha + h) - z_i(x_\alpha)] \cdot [z_j(x_\alpha + h) - z_j(x_\alpha)] \qquad (11)$$

where $z(x_\alpha)$ is the measured value of point x_α, which is decomposed into orthogonal spatial components $z^u(x_a)$ as shows in equation (12) and (13). The parameter $z^u(x_a)$ could be estimated using a linear combination based on the value of n data points in the estimative neighbourhood. Weight coefficients in the linear combination can be determined when the estimated variance is at a minimum in the unbiased condition.

$$z(x_a) = \sum_{u=1}^{N_s} z^u\,(x_a) \qquad (12)$$

$$E\left[z_i^u(x_a) z_j^u(x_a + h)\right] = 0 \quad when \;\; i \neq j \qquad (13)$$

We used an iterative procedure developed by Goulard and Voltz[41] to fit the LMC. The goodness of fit for the LMC is evaluated by the mean error (ME) and mean of squared standardized errors (MSSE) calculated from the cross-validation[40]. ME and MSSE are calculated by equation (14) and (15), respectively.

$$ME = \frac{1}{n} \sum_{\alpha=1}^{n} (z\,(x_a) - z^*(x_a)) \qquad (14)$$

$$MSSE = \frac{1}{n} \sum_{\alpha=1}^{n} \frac{(z(x_a) - z^*(x_a))^2}{\sigma_{KR}^2(x_a)} \qquad (15)$$

where *n* is the number of the data points, $z^*(x_a)$ is the estimated value of point x_a, and $\sigma_{KR}^2(x_a)$ is the standard deviation of estimated value. When the ME is closer to 0 and the MSSE is closer to 1, then the LMC

provides better results.

Second, spatial relationships between various variables at different scales can be analysed using the structure correlation coefficient and principle component analysis (PCA).

The structure correlation coefficient r_{ij}^{u} is the correlation between variables *i* and *j* at the *u*th spatial structure, and it can be calculated using equation (16), where b_{ij}^{u} refers to the element at position (*i, j*) in the co-regionalization matrix B^{u}.

$$r_{ij}^{u} = \frac{b_{ij}^{u}}{\sqrt{b_{ii}^{u} \cdot b_{jj}^{u}}} \qquad (16)$$

The PCA extracts eigenvalues and eigenvectors in the co-regionalization matrix of each scale to calculate the correlation coefficient between variables and principal components at the given scale[39]. Specifically, the PCA decomposes the co-regionalization matrix B^{u} into eigenvectors q_{il} and eigenvalues λ_{l} using equation (17), where Q^{u} is the eigenvector matrix and Λ^{u} is the eigenvalue matrix at the *u*th spatial structure.

$$B^{u} = Q^{u}\Lambda^{u}Q^{uT} = Q^{u}\sqrt{\Lambda^{u}}(Q^{u}\sqrt{\Lambda^{u}})^{T} \qquad (17)$$

Then, correlation coefficient ρ_{il} between the spatial component of the variable and principal component can be calculated by equation (18), where σ^{2} is the variance of the *i*th variable.

$$\rho_{il} = q_{il}\sqrt{\lambda_{l}/\sigma^{2}} \qquad (18)$$

Third, the ordinary cokriging method was used to estimate and map component $z_{i}^{u*}(x)$ and the total variation $z_{i}^{*}(x)$ at each scale. A detailed description of the ordinary cokriging estimation is provided by Goovaerts[42] and Wackernagel[40].

References

1 Wang, R. & Yang, Q. S. Changes of land use and landscape pattern in Taihu Lake Basin (in Chinese). *Chin. J. Appl. Ecology*. **16,** 475-480 (2005).

2 Statistics Bureau of Jiangsu Province. Jiangsu statistical yearbook 2011 (Nanjing, 2011).

3 Shi, X. Z., *et al.* Cross-reference for relating Genetic Soil Classification of China with WRB at different scales. *Geoderma*. **154,** 344-350 (2010).

4 Yan, L. Z., Shi, M. J. & Wang, L. Review of agricultural non-point source pollution in Taihu Lake and Taihu Basin (in chinese). *China Popul. Res. Environ.* **20,** 99-107 (2010).

5 Nelson, E., *et al*. Modeling multiple ecosystem services, biodiversity conservation, commodity production, and tradeoffs at landscape scales. *Front Ecol. Environ.* **7,** 4-11 (2009).

6 Qiu, J. X. & Turner, M. G. Spatial interactions among ecosystem services in an urbanizing agricultural watershed. *P. Natl. Acad. Sci. USA.* **110,** 12149-12154 (2013).

7 Li, H. P., Yang, G. S., Huang, W., Li, Z. F. & Jin, Y. Simulating fluxes of non-point source nitrogen from upriver region of Taihu Basin (in Chinese). *Acta Pedologica Sinica.* **44,** 1063-1069 (2007).

8 Ying, L. L., Hou, X. Y., Lu, X. & Zhu, M. M. Discussion on the export coefficient method in non-point source pollution studies in China (in Chinese). *J. Water Res.& Water Eng.* **21,** 90-95 (2010).

9 Nelson, E., *et al.* InVEST User's Guide (eds. Sharp, R, *et al*) 131-141 (Natural capital project, 2012).

10 Liu, M., Huang, G. H., Liao, R. F., Li, Y. P. & Xie, Y. L. Fuzzy two-stage non-point source pollution management model for agricultural systems: A case study for the Lake Tai Basin, China. *Agr. Water Manage.* **121,** 27-41 (2013).

11 Li, H. P., Huang, W. Y., Yang, G. S. & Liu, X. M. Non-point pollutant concentrations for different land uses in Lihe River watershed of Taihu Region (in Chinese). *China Environ. Sci*. **26,** 243-247 (2006).

12 Jin, Y., Li, H. P. & Li, J. L. The impact of non- point pollutant load of land-use changes in Taihu Basin (in Chinese). *J. Agro-Environ. Sci.* **26,** 1214- 1218 (2007).

13 Li, Y. & Li, H. P. Influence of landscape characteristics on non-point source pollutant output in Taihu Upper-River Basin (in Chinese). *Environ. Sci.* **29,** 1319-1324 (2008).

14 Dennedy-Frank, P. J., Muenich, R. L., Chaubey, I. & Ziv, G. Comparing two tools for ecosystem service assessments regarding water resources decisions. *J. Environ. Manage.* **177,** 331-340 (2016).

15 Yao, Y. J., *et al.* Estimation of the terrestrial water budget over northern China by merging multiple datasets. *J. Hydrol.* **519,** 50-68 (2014).

16 Zhang, L., *et al*. A rational function approach for estimating mean annual evapotranspiration. *Water Res. Res.* **40,** 89-97 (2004).

17 Canadell, J., *et al.* Maximum rooting depth of vegetation types at the global scale. *Oecologia.* **108,** 583-595 (1996).

18 Huang, L., *et al.* Root distribution in the different forest types and their relationship to soil properties (in Chinese). *Acta Ecol. Sinica.* **32,** 6110-6119 (2012).

19 Liu, Y., Cai, L. G., Fernando, R. M. & Pereira, L. S. Calculation method of the soil water upword flux at the botton boundary of the root zone (in Chinese). *J. Hydraul. Eng*. **32,** 19-25 (2001).

20 Lv, G., Wei, Z. P., Gao, Y. X. & Li, H. W. Study on relationship between plant roots and soil anti-erodibility of different land utilization types (in Chinese). *Agr. Res. Arid Area.* **31,** 111-115 (2013).

21 Gao, J. F. & Wen, Y. H. Impact of Land Use Change on Runoff of Taihu Basin (in Chinese). *Acta Geogr.* Sinica. **57,** 194-200 (2002).

22 Ji, D., *et al.* The response relationship between underlying surface changing and climate change in the Taihu Baisn (in chinese). *J. Nat. Res.* **28,** 51-62 (2013).

23 Lv, W., S., Yang, G., Wan, R. R. & Zhu, Q. Comparison study on evapotranspiration characteristics of different landuse types in Taihu Lake Watershed (in Chinese). *Bull. Soil Water Conserv.* **22,** 202-209 (2013).

24 Sánchez-Canales, M., *et al*. Sensitivity analysis of ecosystem service valuation in a Mediterranean watershed. *Sci. Total Environ.* **440,** 140-153 (2012).

25 Fu, B. J., *et al.* Assessment of soil erosion at large watershed scale using RUSLE and GIS: A case study in the Loess Plateau of China. *Land Degrad. Dev.* **16,** 73-85 (2005).

26 Jia, X. Q., *et al.* The tradeoff and synergy between ecosystem services in the Grain-for-Green areas in Northern Shaanxi, China. *Ecol. Indic.* **43,** 103-113 (2014).

27 Wischmeier, W. H. & Smith, D. D. Predicting rainfall erosion losses: A guide to conservation planning. (United States Department of Agriculture, 1978).

28 Williams, J., Renard, K. & Dyke, P. EPIC: A new method for assessing erosion's effect on soil productivity. *J. Soil Water Conserv.* **38,** 381-383 (1983).

29 Liu, B. Y., Nearing, M. A., Shi, P. J. & Jia, Z. W. Slope length effects on soil loss for steep slopes. *Soil Sci. Soc. Am. J.* **64,** 1759-1763 (2000).

30 Lv, J. S., Wu, Q. Y., Zhang, Z. L. & Liu, Y. Land Use Change and Ecological Security Assessment in Jining City Based on RS and GIS (in Chinese). *Sci. Geogr. Sinica.* **32,** 928-935 (2012).

31 Zhang, L. & Meng, Y. L. Evaluation of water and soil loss in Taihu Basin of Jiangsu Province based on GIS (in Chinese). *Acta Agr. Jx*. **21,** 129-132 (2009).

32 Zhang, Y., Zhang, H., Peng, B. Z. & Yang, H. Soil erosion and nutrient loss of various land use patterns (in Chinese). *Bull. Soil Water Conserv.* **23,** 23-27 (2003).

33 Zheng, H. A., Wu, J. L. & Lin, L. Using ^{137}Cs tracer technology to inverstigate soil erosion distribution and total erosion amount in Taihu Lake Catchment (in Chinese). *Mar. Geol.Quaternary Geol.* **28,** 79-85 (2008).

34 Lieth, H. & Box, E. Evapotranspiration and primary productivity: CW Thornthwaite memorial model. *Publ. in Climatol.* **25,** 37-46 (1972).

35 LUMD, LCRC, CAU, BNU & HNU. Regulations of Farmland Grading (in Chinese). (Ministry of Land and Resources of P. R. C., 2003).

36 OLR, J. S. Technical proposal of accounting arable land production capacity in Jiangsu Province (in Chinese) (Land Resources Department of Jiangsu Province, 2008).

37 Grimm, N. B., *et al.* Climate-change impacts on ecological systems: introduction to a US assessment. *Front. Ecol. Environ.* **11,** 456-464 (2013).

38 Yan, Q. W., Bing, Z. F. & Zhao, H. A. Method of Spatialization of Population Density (in Chinese). *Geogr Geo-Inform. Sci.* **21,** 45-48 (2005).

39 Lv, J. S., Liu, Y., Zhang, Z. L. & Dai, J. R. Factorial kriging and stepwise regression approach to identify environmental factors influencing spatial multi-scale variability of heavy metals in soils. *J. Hazard. Mater.* **261,** 387-397 (2013).

40 Wackernagel, H. Multivariate geostatistics: an introduction with applications (Springer-Verlag, 2003).

41 Goulard, M. & Voltz, M. Linear co-regionalization model: tool for estimation and choice of cross-variogram matrix. *Math. Geosci.* **24,** 269-286 (1992).

42 Goovaerts, P. Geostatistics for natural resources evaluation (Oxford University Press, 1997).

Table S1 List of data inputs and parameters used in water purification services mapping

Code	Data inputs	Unit	Data format
1	Digital elevation model (DEM)	m	30 m GIS raster dataset
2	Root restricting layer depth (Rest.layer.depth)	mm	100 m GIS raster dataset
3	Precipitation (P)	mm	30 m GIS raster dataset
4	Plant available water content (PAWC)	mm	100 m GIS raster dataset
5	Annual average potential evapotranspiration (ET_0)	mm	30 m GIS raster dataset
6	Land use/land cover	--	100 m GIS raster dataset
7	Maximum root depth for vegetated land use classes	mm	floating point values
8	The plant evapotranspiration coefficient for each LULC class	--	floating point values
9	The nitrogen export coefficient for each land use	kg/ha	floating point values
10	The phosphorus export coefficient for each land use	kg/ha	floating point values
11	The nitrogen filtration efficiency for each land use	%	floating point values
12	The phosphorus filtration efficiency for each land use	%	floating point values
13	Threshold flow accumulation value	--	floating point values

Table S2 List of data inputs and parameters used in water supply service mapping

Code	Data inputs	Unit	Data format
1	Root restricting layer depth (Rest.layer.depth)	mm	100 m GIS raster dataset
2	Precipitation (P)	mm	30 m GIS raster dataset
3	Plant available water content (PAWC)	mm	100 m GIS raster dataset
4	Annual average potential evapotranspiration (ET_0)	mm	100 m GIS raster dataset
6	Land use/land cover	--	100 m GIS raster dataset
7	Maximum root depth for vegetated land use classes	mm	floating point values
8	The plant evapotranspiration coefficient for each LULC class	--	floating point values
9	Z parameter	--	floating point values

Table S3 List of data inputs and parameters used in soil retention service mapping

Code	Data inputs	Unit	Data format
1	Digital elevation model (DEM)	m	30 m GIS raster dataset
2	Rainfall erosivity index (R)	MJ·mm/(ha·h·yr)	30 m GIS raster dataset
3	Soil erodibility (K)	t·ha·h/(ha·MJ·mm)	30 m GIS raster dataset
4	Slope length and steepness index (LS)	--	30 m GIS raster dataset
6	Cover-management factor (C)	--	floating point values
7	Support practice factor (P)	--	floating point values
8	Land use/land cover	--	100 m GIS raster dataset
9	Threshold flow accumulation value	--	floating point values

Table S4 List of data inputs and parameters used in crop production service mapping

Code	Data inputs	Unit	Data format
1	Precipitation (P)	mm	30 m GIS raster dataset
2	Annual average evapotranspiration (ET_0)	mm	30 m GIS raster dataset
3	Annual average temperature (T)	℃	30 m GIS raster dataset
4	Cultivated land quality data	--	100 m GIS raster dataset
5	Land use/land cover	--	100 m GIS raster dataset

Table S5 List of 31 influence factors used in analysis

Factor type	Factors code	Description	Original resolution	Unit
**Physical environmental factors**				
	SUN	Percentage of actual and potential sunshine duration	Point	%
Climate	ARH	Annual average relative humidity	Point	%
	TEM	Annual average temperature	Point	℃
Terrain	ALT	Altitude	30 m	m
	SLOP	Slope	30 m	in degrees
Hydrology	WATER	Water density	1 km	ha/km^2
Soil	TN	Soil total nitrogen content at 0–20cm depth	100 m	mg/m^3
	TP	Soil total phosphorous content soil at 0–20cm depth	100 m	mg/m^3
	TK	Soil total Potassium content at 0–20cm depth	100 m	mg/m^3
	SOM	Soil organic matter content at 0–20cm depth	100 m	mg/m^3
	BULK	Bulk density	100 m	g/cm^3
	SAND	Sand content of the soil at 0–20cm depth	100 m	(%)
	SILT	Silt content of the soil at 0–20cm depth	100 m	(%)
	CLAY	Clay(< 0.002mm) content of the soil at 0–20cm depth	100 m	(%)
NDVI	NDVI	Normalized difference vegetation index	30 m	[-1,1] extent
	DISWAT	Euclidean distance to water	30 m	m
Accessibility	DISURB	Euclidean distance to urban areas	30 m	m
	DISVILL	Euclidean distance to villages	30 m	m
**Social-economic factors**				
Development	POP	Population density	1 km	inhab./km^2
	GDP	Gross domestic production per unit area	1 km	thousand dollar/km^2
	URBENS	Urban density	1 km	ha/km^2
Residence	VILLDENS	Village density	1 km	ha/km^2
	ROADENS	Road density	1 km	km/km^2
Agriculture	AGRIPOP	Agricultural population density	1 km	farmer/km^2
	AGRIGDP	Agricultural output value per unit area	1 km	thousand dollar/km^2
	AGRPOWER	Total power of agricultural machinery	1 km	kilowatt/ha
	FARINCM	Farmer's annual average income	1 km	dollar/farmer
Land use	ARABLE	Arable area ratio of per km^2	1 km	%
	FOREST	Forest area ratio of per km^2	1 km	%
	GRASS	Grassland area ratio of per km^2	1 km	%
	WETLAND	Wetland area ratio of per km^2	1 km	%

Table S6 Summary of descriptive statistics for each ecosystem service

Ecosystem Service	Unit	Observation number	Min	Max	Mean	SD
Nitrogen loading	kg/ha	10000	0	62.15	12.39	8.96
Phosphorus loading	kg/ha	10000	0	6.51	1.08	0.88
Water supply	mm/y	10000	197.09	1173.16	602.48	176.77
Soil retention	t/ha	10000	0	82.54	1.86	3.75
Crop production	t/ha	10000	0	16.42	3.61	3.15

Table S7 Parameter of the LMC fitted and the percentages of total variation at each scale

Ecosystem service	Parameters of LMC analysis			Percentage of total variation at each scale (%)		
	Nugget effect	Sill of short-range	Sill of long-range	Nugget effect	Short-range (local) scale	Long-range (regional) scale
Nitrogen loading	0.5807	0.1619	0.3206	54.62	15.23	30.15
Phosphorus loading	0.5673	0.1816	0.3562	51.33	16.44	32.23
Water supply	0.3607	0.1161	0.6175	32.96	10.61	56.43
Soil retention	0.8802	0.0488	0.0549	89.46	4.96	5.58
Crop production	0.6560	0.136	0.1643	68.60	14.22	17.18

Table S8 Result of Principle Component Analysis (PCA) at each scale

Ecosystem service	Classical PCA		Local scale		Regional scale	
	F1	F2	F1	F2	F1	F2
Nitrogen loading	0.913	-0.108	0.669	0.581	0.860	0.317
Phosphorus loading	0.771	-0.442	0.896	-0.307	0.781	0.451
Water supply	0.799	-0.072	0.985	-0.108	0.656	-0.480
Soil retention	0.292	0.703	0.493	-0.459	0.944	-0.157
Crop production	0.741	0.339	0.455	0.764	0.934	-0.042
Eigen value	2.444	0.997	0.397	0.185	1.180	0.283
Ratio of variance	48.87	19.94	62.59	29.14	78.51	18.80

Table S9 Regression models for nutrient loadings at the local scale

Nitrogen loading					Phosphorous loading				
Factor	Coef	SE	Sig.	R^2	Factor	Coef	SE	Sig.	R^2
ARABLE	0.220	0.022	0.000	0.237	URBENS	0.001	0.000	0.000	0.417
AGRIGDP	7.607E-05	0.001	0.000	0.186	DISURB	-5.535E-06	0.000	0.003	0.209
FARINCM	2.647E-06	0.001	0.008	0.158	POP	1.412E-05	0.000	0.000	0.205
WATER	-0.002	0.000	0.000	0.152	ARABLE	-0.145	0.021	0.000	0.166
VILLDENS	-0.002	0.000	0.000	0.151	DISWAT	2.286E-05	0.000	0.000	0.155
SOM	-0.062	0.005	0.000	0.135	GDP	8.052E-06	0.000	0.000	0.154
DISVILL	-6.148E-05	0.000	0.000	0.132	DISVILL	2.514E-05	0.000	0.004	0.148
GDP	-4.783E-06	0.000	0.000	0.108	WATER	-0.003	0.000	0.000	0.141
BULK	0.104	0.018	0.000	0.091	AGRIGDP	-9.826E-05	0.000	0.018	0.163
POP	-1.072E-05	0.001	0.038	0.086	ROADENS	8.095E-05	0.000	0.000	0.139
NDVI	0.211	0.025	0.000	0.036	SOM	-0.032	0.005	0.000	0.134
TEM	0.067	0.017	0.000	0.031	ARH	0.036	0.005	0.000	0.114
SUN	0.035	0.007	0.000	0.019	FARINCM	-1.650E-06	0.000	0.044	0.102
ROADENS	-4.104E-05	0.000	0.006	0.016	WETLAND	-0.754	0.265	0.005	0.056
ALT	-0.017	0.003	0.005	0.010	GRASS	-0.597	0.261	0.022	0.054
DISWAT	2.286E-05	0.000	0.000	0.007	TEM	-0.079	0.022	0.000	0.015
AGRPOWER	-0.002	0.001	0.001	0.001	SUN	-0.019	0.006	0.002	0.014
(Constant)	-2.680	0.049	0.000		SLOP	-0.003	0.001	0.007	0.011
					(Constant)	-0.447	0.405	0.270	

Table S10 Regression models for nutrient loadings at the regional scale

Nitrogen loading					Phosphorous loading				
Factor	Coef	SE	Sig.	R^2	Factor	Coef	SE	Sig.	R^2
WATER	-0.004	0.000	0.000	0.429	WATER	-0.003	0.000	0.000	0.378
SUN	0.022	0.006	0.000	0.301	SUN	0.063	0.006	0.000	0.308
NDVI	0.083	0.023	0.004	0.213	SOM	0.056	0.005	0.000	0.295
DISVILL	-2.820E-05	0.000	0.001	0.248	DISURB	-1.217E-05	0.000	0.000	0.274
TN	0.097	0.024	0.000	0.208	NDVI	-0.041	0.027	0.006	0.201
SOM	0.049	0.005	0.000	0.176	TEM	-0.076	0.016	0.000	0.137
TEM	-0.276	0.023	0.000	0.158	DISWAT	1.524E-05	0.000	0.000	0.136
BULK	-0.063	0.017	0.000	0.124	BULK	-0.077	0.018	0.000	0.128
FOREST	-0.326	0.036	0.000	0.121	FOREST	-0.377	0.039	0.000	0.123
DISWAT	1.130E-05	0.000	0.003	0.114	SLOP	-0.003	0.001	0.036	0.118
ALT	-0.001	0.000	0.000	0.110	TK	-0.025	0.008	0.001	0.109
VILLDENS	0.003	0.001	0.003	0.098	TP	0.101	0.023	0.000	0.101
AGRIGDP	3.047E-06	0.000	0.001	0.096	ROADENS	1.488E-05	0.000	0.026	0.101
DISURB	-6.068E-06	0.000	0.001	0.083	AGRPOWER	-0.001	0.001	0.011	0.074
TP	-0.731	0.170	0.000	0.021	POP	3.240E-06	0.000	0.008	0.073
GRASS	-0.828	0.256	0.001	0.021	URBDENS	0.002	0.001	0.039	0.043
GDP	-8.383E-07	0.000	0.035	0.021	ALT	-0.002	0.000	0.000	0.018
SLOP	-0.002	0.001	0.103	0.019	GRASS	-0.915	0.173	0.001	0.013
ARH	0.062	0.005	0.000	0.017	WETLAND	-0.612	0.176	0.027	0.010
ROADENS	3.684E-05	0.000	0.022	0.006	ARH	0.200	0.029	0.030	0.008
(Constant)	-0.302	0.413	0.046		(Constant)	-1.033	0.465	0.026	

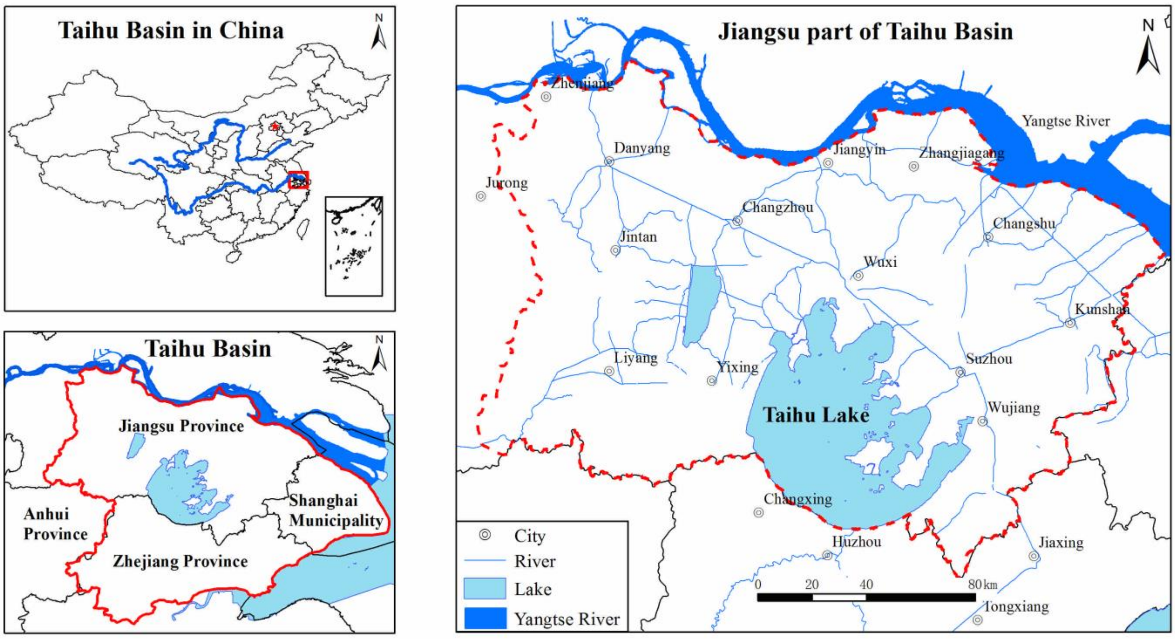

Figure S1 Location of the study area. Administrative division data of different levels were obtained from the National Earth System Science Data Sharing Infrastructure (http://www.geodata.cn). Water data were provided by the Nanjing Institute of Geography & Limnology, Chinese Academy of Sciences. Maps generated with ArcGIS 10.2.2 (http://www.esri.com/software/arcgis).

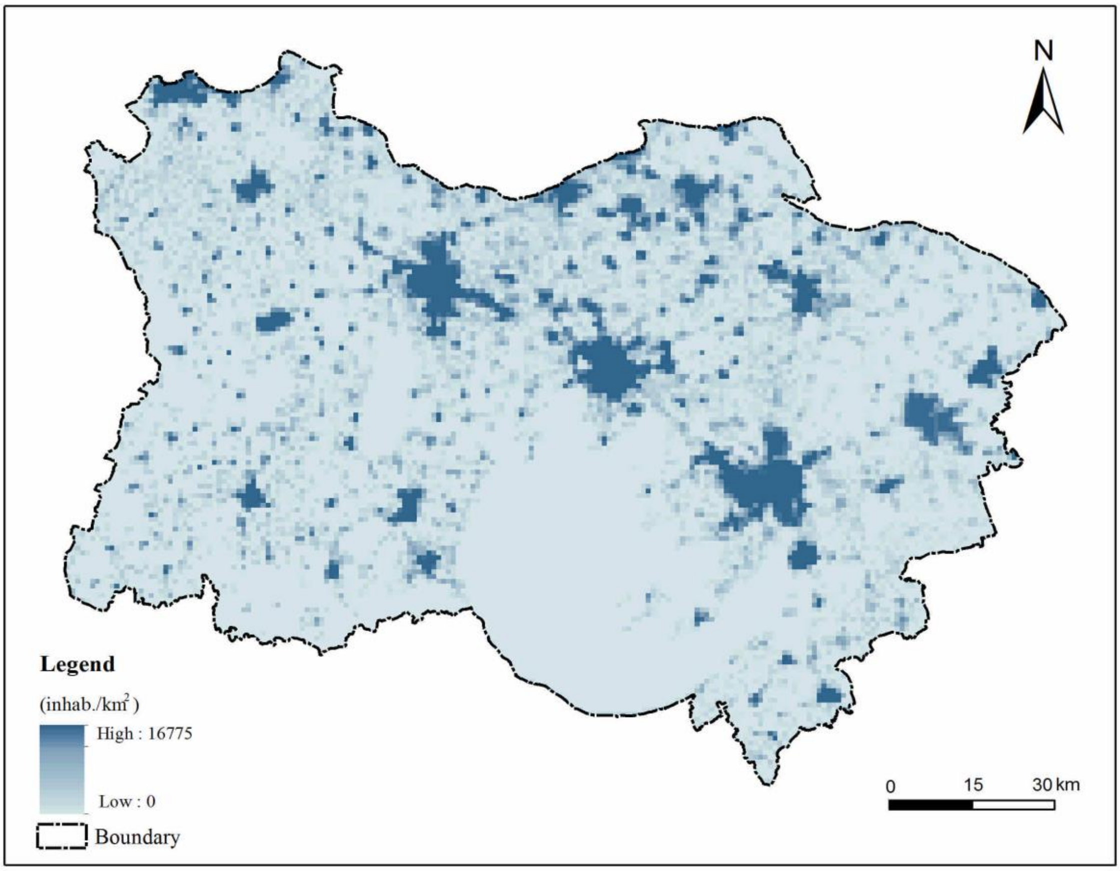

Figure S2 Population density of the study area. Original data were provided by the Institute of Geographic Science and Natural Resources Research, Chinese Academy of Sciences. Maps generated with ArcGIS 10.2.2 (http://www.esri.com/software/arcgis).

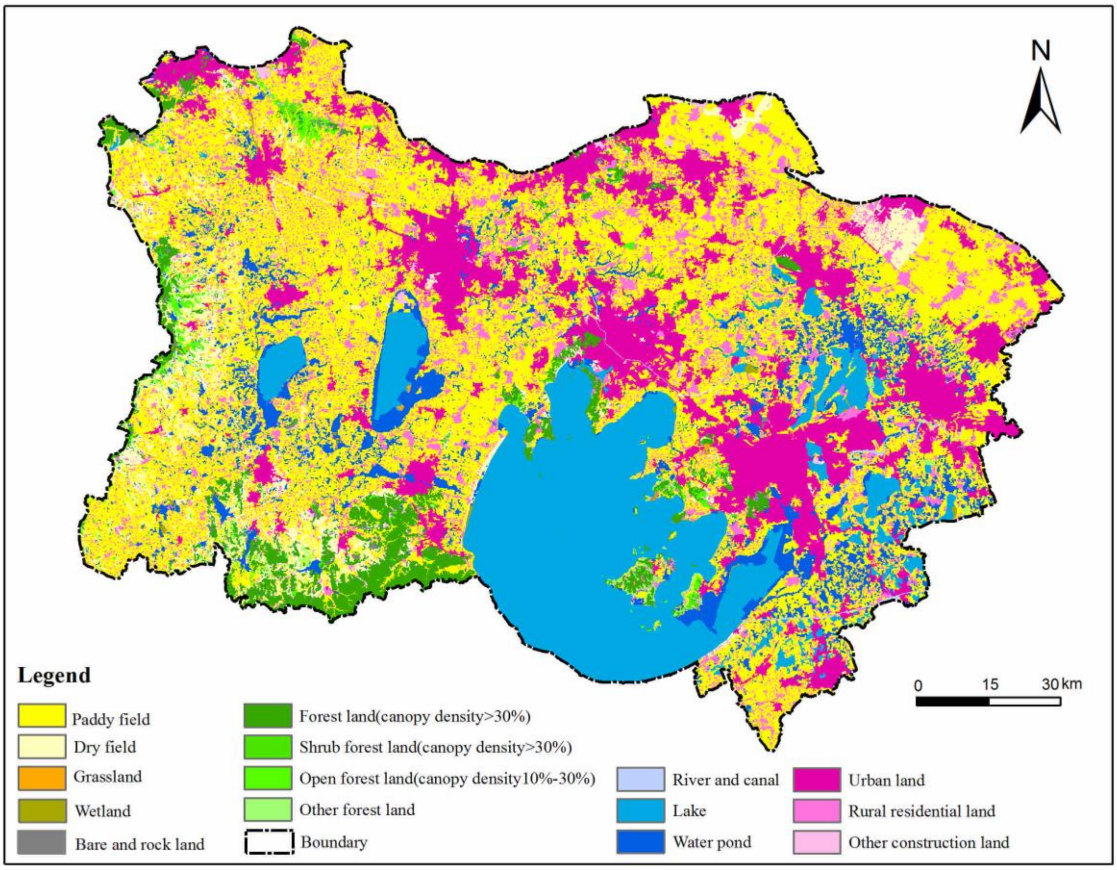

Figure S3 Land use/land cover of the study area. Original data were obtained from the National Earth System Science Data Sharing Infrastructure (http://www.geodata.cn). Maps generated with ArcGIS 10.2.2 (http://www.esri.com/software/arcgis).

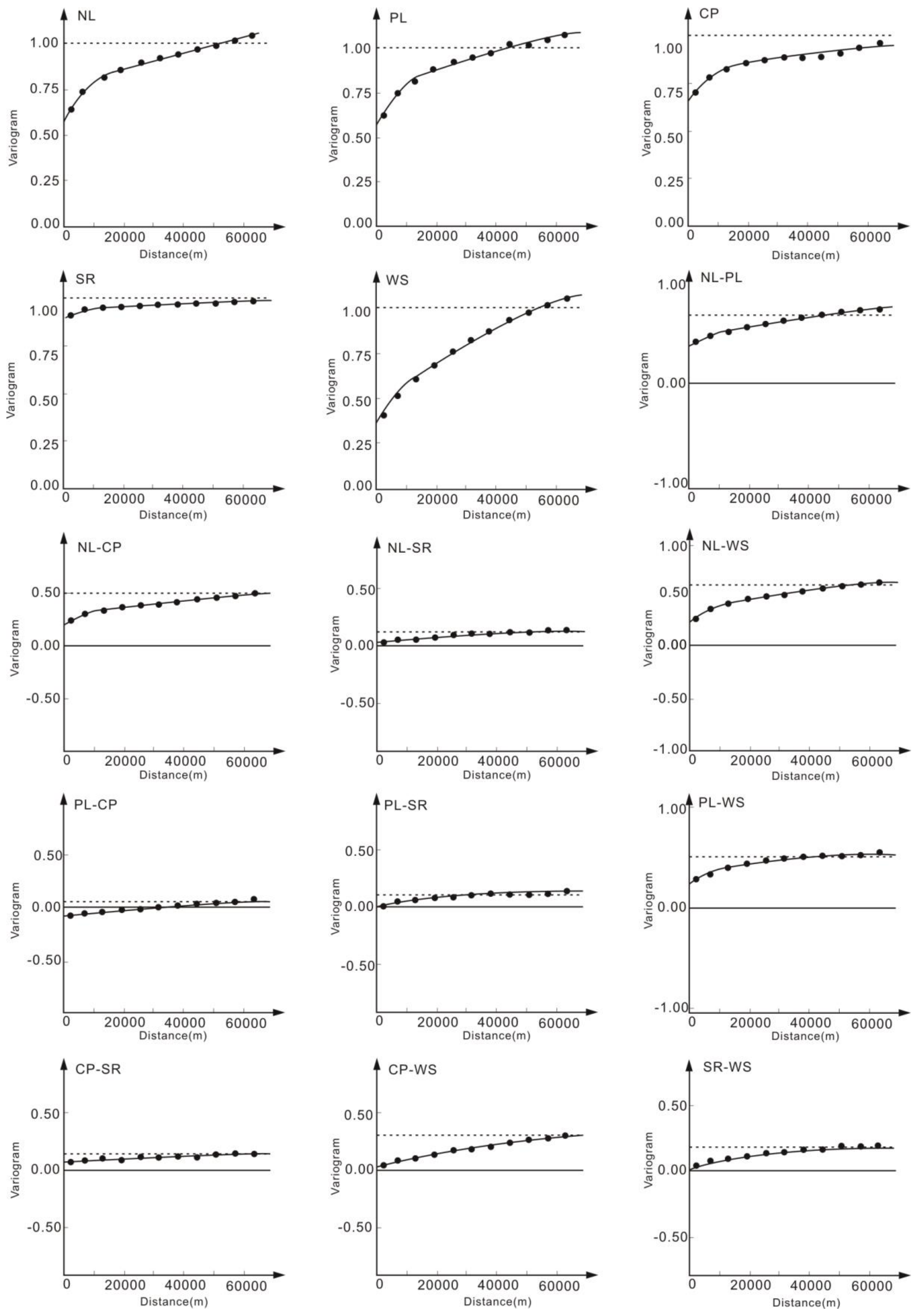

Figure S4 Experimental simple and cross-experimental variograms (dots) with the linear model of co-regionalization (solid lines). Dashed lines are total variances.
